# Validation of the Chinese version of the FOUR score in the assessment of neurosurgical patients with different level of consciousness

**DOI:** 10.1186/s12883-015-0508-9

**Published:** 2015-12-10

**Authors:** Juan Peng, Yingying Deng, Fangyao Chen, Xiaomei Zhang, Xiaoyan Wang, Ying Zhou, Hongzhen Zhou, Binghui Qiu

**Affiliations:** Department of Nursing, Nanfang Hospital, Southern Medical University, Guangdong, 510515 China; Department of Neurosurgery, Nanfang Hospital, Southern Medical University, Guangdong, 510515 China; Department of Biostatistics, School of Public Health and Tropical Medicine, Southern Medical University, Guangdong, 510515 China; Department of Neurology, Nanfang Hospital, Southern Medical University, Guangdong, 510515 China; Intensive Care Unit, Cancer Center of Guangzhou Medical University, Guangdong, 510095 China

**Keywords:** Full outline of un-responsiveness score, Prognosis, Glasgow coma scale

## Abstract

**Background:**

The Glasgow Coma Scale (GCS) is currently the most widely used scoring system for comatose patients. A decade ago, the Full Outline of Unresponsiveness (FOUR) score was devised to better capture four functional aspects of consciousness (eye, motor responses, brainstem reflexes, and respiration). This study aimed to validate the Chinese version of the FOUR score in patients with different levels of consciousness.

**Methods:**

The study had two phases: (1) translation of the FOUR score, and (2) assessment of its reliability and validity. The Chinese version of the FOUR score was developed according to a standardized protocol. One hundred-twenty consecutive patients with acute brain damage, admitted to Nanfang Hospital (Southern Medical University, Guangdong, China) from November 2014 to February 2015, were enrolled. The inter-rater agreement for the FOUR score and GCS was evaluated using intraclass correlation coefficient (ICC). Receiver operating characteristic (ROC) curves were established to determine the scales’ abilities to predict outcome.

**Results:**

The rater agreement was excellent both for FOUR (ICC = 0.970; *p* < 0.001) and GCS (ICC = 0.958; *p* < 0.001). The FOUR score yielded an excellent test-retest reliability (ICC = 0.930; *p* < 0.001). Spearman’s correlation coefficients between GCS and the FOUR score were high: *r* = 0.932, first rating; *r* = 0.887, second rating (all *p* < 0.001). Areas under the curve (AUC) for mortality were 0.834 (95 % CI, 0.740–0.928) and 0.815 (95 % CI, 0.723–0.908) for the FOUR score and GCS, respectively.

**Conclusions:**

The Chinese version of the FOUR score is a reliable scale for evaluating the level of consciousness in patients with acute brain injury.

## Background

The Glasgow Coma Scale (GCS) is a widely used tool to measure objectively the patient’s level of consciousness (LOC) in the clinical setting. However, the GCS has a few limitations [1, 2]. First, it cannot detect subtle clinical changes in comatose patients due to the lack of important clinical indicators such as brainstem reflexes and respiration pattern (including mechanical ventilation), which reflect the consciousness level [3]. In addition, for intubated patients, the GCS cannot properly assess the verbal component, and scoring difficulties have been displayed by unexperienced nurses and paramedics [1]. Most importantly, a 10-year retrospective study revealed that the GCS cannot predict the outcome of patients with traumatic brain injury (TBI) [2]. Therefore, other scales are being developed for this purpose, but most of them are not widely accepted because of their complexity and non-reliability [4–7].

A novel coma scaling system, the Full Outline of Un-Responsiveness (FOUR) score was developed by the Mayo Clinic in 2005 [3]. It evaluates four functional categories: eye response, motor response, brainstem reflexes, and respiration pattern (including mechanical ventilation). All the four categories are scored from 0 to 4 points, with 4 representing normal, and 0 indicating no function [3]. Patients are considered as brain dead with an overall score of 0 [8]. Finally, this score has also been recommended by the latest guidelines of the European Society of Intensive Care Medicine (ESICM) [9]. Previous studies compared the FOUR score to the GCS score and showed that they were comparable [10–14].

Recently, several prospective studies have introduced and validated the FOUR score as a reliable tool for the assessment of patients in medical intensive care units, neuro-intensive care units, and neurology and emergency departments [10, 11, 15–17]. In addition, the FOUR score has been used to assess cirrhotic [18] and pediatric [19] patients. It is also valid in predicting the outcome of patients after cardiac arrest [20] and traumatic brain injury [21]. Interestingly, the FOUR score has already been translated into many languages such as Italian [12], French [22], Spanish [11], Korean [23], and Turkish [24], but no Chinese version is available to date.

Therefore, this study had two aims: 1) the translation of the FOUR score into Chinese; and 2) the validation of the FOUR score as a measure of the level of consciousness.

## Methods

### Development of the Chinese version of the FOUR Score

**Fig. 1:**
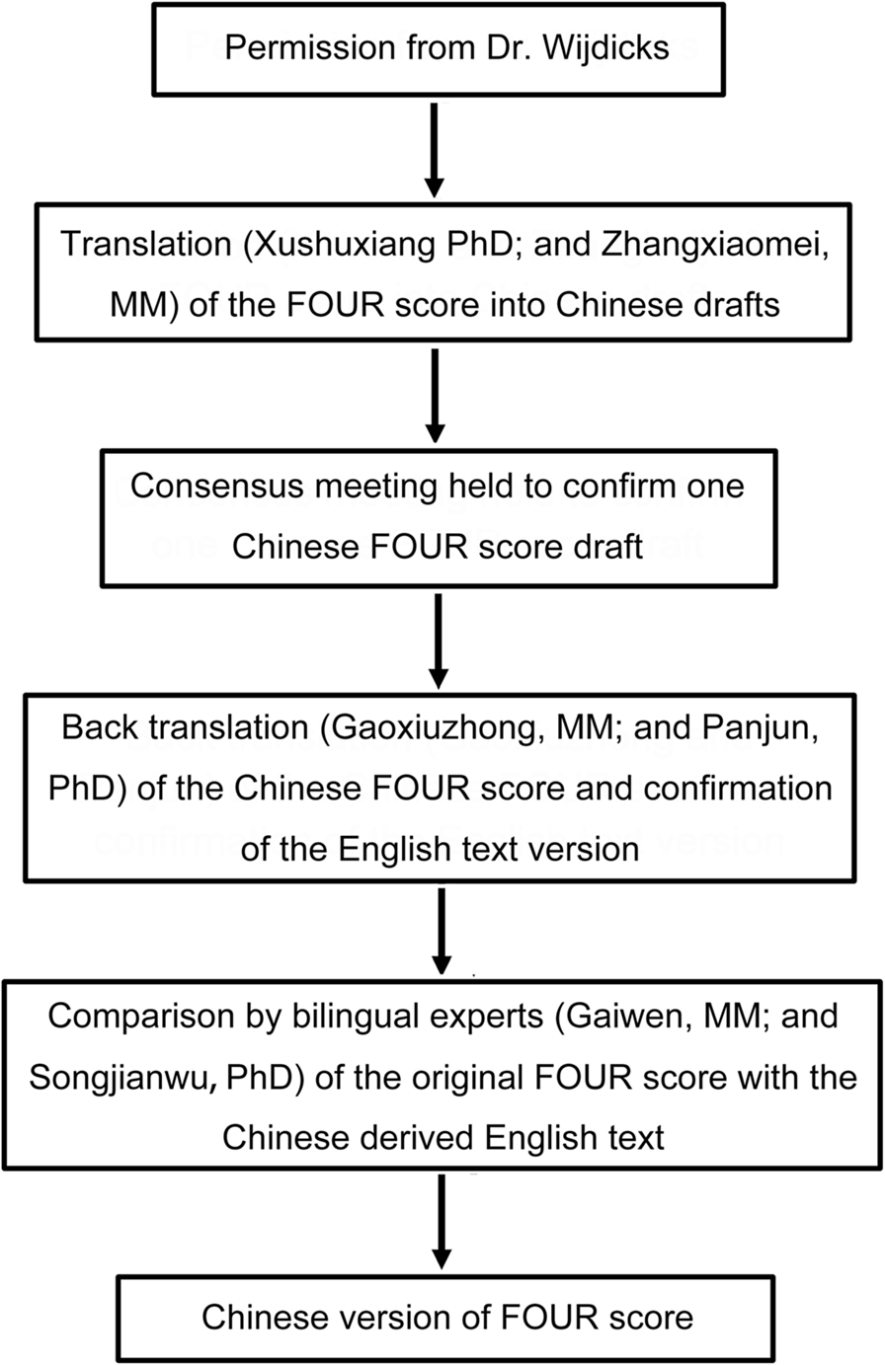
Translated Process of the Chinese version of Four score

The translation process is displayed in Fig. 1. In the translation process, we improved the following points to allow the medical staff to apply the FOUR score more easily in Chinese. First, for the eye response, the translation resulted in “the eyelids closed but open to loud voice, and eyelids closed but open to pain”, which are misunderstood as patients eyes open to loud voice or pain; this omitted important information that patients eyelids are closed in most cases, but open to loud voice or pain. Therefore, a more specific and clear translation was provided for this point. Secondly, for the motor response, the author originally thought that focus should be on observing patients’ arm response, but this point is not reflected in the original English language text. Therefore, we added arm flexion or extension response to pain in order to be more accurate and understood by the medical staff. Finally, the Chinese version of the FOUR score was approved by the consensus meeting. In conclusion, the Chinese version of the FOUR score was validated (Tables 1 and 2).

**Table 1:** Chinese version of the FOUR SCORE

FOUR Score	
Eye response	Brainstem reflexes
4: eyelids open or opened, tracking, or blinking to command	4: pupil and corneal reflexes present
3: eyelids open but not tracking	3: one pupil wide and fixed
2: eyelids closed but open to loud voice	2: pupil or corneal reflexes absent
1: eyelids closed but open to pain	1: pupil and corneal reflexes absent
0: eyelids remain closed with pain	0: absent pupil, corneal, and cough reflex
Motor response	Respiration
4: thumbs-up, fist, or peace sign	4: not intubated, regular breathing pattern
3: localizing to pain	3: not intubated, Cheyne–Stokes breathing pattern
2: flexion of the upper limbs response to pain	2: not intubated, irregular breathing
1: extension of the upper limbs response to pain	1: breathes above ventilator rate
0: no response to pain or generalized myoclonus status	0: breathes at ventilator rate or apnea

**Table 2:** in Chinese

无反应性全面量表	
眼睛反应	脑干反射
4分:眼睑张开或被张开,且眼球跟随物体移动,或随指令眨眼	4分:瞳孔和角膜反射均存在
3分:眼睑张开但眼球不跟随移动	3分:一侧瞳孔放大和固定
2分:眼睑闭合但大声呼唤时睁眼	2分:瞳孔或角膜反射其中一项消失
1分:眼睑闭合但疼痛刺激时睁眼	1分:瞳孔和角膜反射均消失
0分:疼痛刺激时仍保持眼睑闭合	0分:瞳孔反射、角膜反射和咳嗽反射均消失
运动反应	呼吸
4分:可竖起拇指、握拳或做V字手势	4分:无气管插管,呼吸节律规则
3分:疼痛刺激时能定位	3分:无气管插管,潮式呼吸
2分:疼痛刺激时上肢屈曲	2分:无气管插管, 呼吸不规则
1分:疼痛刺激时上肢伸直	1分:呼吸频率大于机械通气频率
0分:疼痛刺激时无反应或全身呈肌阵挛状态	0分:呼吸机控制呼吸或呼吸暂停

### Reliability and validity assessment of the Chinese version of the FOUR score

#### Patients

This study was approved by the ethical committee of the Nanfang Hospital, Southern Medical University, Guangdong, China (No. NFEC-2014-124); informed consent was obtained from all patients or their caregivers. A total of 120 consecutive patients with acute brain-damage were enrolled from November 2014 to February 2015 at our Neurosurgical intensive care unit and evaluated with the GCS and FOUR scores on the day of admission. Adult patients >18 years old diagnosed with acute traumatic brain injury or non-TBI (intracerebral hemorrhage, subarachnoid hemorrhage, brain tumor, hydrocephalus, epilepsy, cerebral infarction, etc.) were recruited. Exclusion criteria were: 1) treatment with neuromuscular junction blockers or sedatives; 2) hemodynamic instability (systolic blood pressure [BP] <80 mm Hg); or 3) substance or alcohol abuse. Demographic data, vital signs, diagnosis, day of evaluation, and degree of consciousness (awake or alert, drowsy, stuporous, or comatose, according to Ropper [25]), were recorded.

#### Procedure

Patients were assessed by two neurosurgery residents (R/R) or nurses (N/N), or a combination of a resident and a nurse (R/N); each health care professional had more than 10 years of clinical experience in a neurosurgical\neurological intensive care unit (ICU), and patients were assessed by a randomly chosen rater pair. For intubated patients, the lowest GCS verbal score was considered to be 1. Raters watched a 20-min videos with patient examples and instructions about the FOUR score. Subsequently, a one-page handout with written instructions describing both the FOUR score and GCS were provided to raters who were given opportunities to assess patients before study beginning. In addition, in order to minimize the possible changes in patient’s level of consciousness, all the assessments were completed within one hour. In addition, 30 randomly selected patients were evaluated by the FOUR score on the second day of hospitalization to test the test-retest reliability.

#### Outcome assessment

Patients’ in-hospital mortality and clinical diagnosis of brain death were documented. Outcome was assessed at 3 months using the modified Rankin Scale (MRS) [26], which assesses the patients’ overall function and mortality. Simply, a score of 0 indicates no symptoms; 1 represents no evident disability despite symptoms; 2 indicates slight disability, with no ability to carry out all routine activities, ability to take care of own affairs; 3 represents moderate disability, requiring some help, but ability to walk without assistance; 4 indicates moderately severe disability, with no ability to walk and attend to own bodily needs without assistance; 5 represents severe disability, e.g. in bedridden patients with incontinence, requiring constant nursing care; 6 indicates death. In this study, a MRS score between 0 and 2 indicated a good recovery for the patient; a poor outcome was reflected by a score between 3 and 6.

#### Statistical analyses

SPSS 13.0 (SPSS Inc., Chicago, IL, USA) was used for data analysis. Data normality was analyzed using the Kolmogorov-Smirnov test. Normally distributed continuous data are expressed as mean ± standard deviation (SD). Non-normally distributed continuous data are presented as median (range). Categorical variables are presented as frequencies. For the FOUR score scales, intraclass correlation coefficient (ICC) was used to measure the inter-rater agreement and test-retest reliability. Cronbach’s α and Spearman’s correlation coefficients were estimated to assess internal consistency and construct validity (with the GCS as criterion). To compare the FOUR score and GCS for prediction of in-hospital mortality and 3-month MRS 3-6, prognostic performance was evaluated by receiver operating characteristic (ROC) curves and areas under the curve (AUC). In general, an AUC of 1.0 refers to a perfect test, while a perfectly inaccurate test has an AUC of 0.0. Usually, an AUC higher than 0.75 indicates that the predictors of the scale have moderate discriminative properties, while predictors are excellent with an AUC ≥0.90. The best cut-off point was chosen to yield the maximum Youden index [27]. Two-tailed *P* < 0.05 was considered statistically significant.

## Results

### Characteristics of the patients

Detailed patients’ characteristics are summarized in Table 3. Three patients dropped out, among which the MRS score could not be obtained at the 3-month telephone follow-up for two patients, while the other patient used sedatives during the evaluation and had to be was excluded. The characteristics of these three patients were: 1) male; drowsiness on hospitalization; 58 years old; subarachnoid hemorrhage; no mechanical ventilation; FOUR score: 13; GCS score: 11; 2) male; light coma on hospitalization; 34 years old; cerebral hemorrhage; no mechanical ventilation; FOUR score: 11; GCS: 7; and 3) this patient used sedatives during the 3-month evaluation; female; deep coma on hospitalization; 43 years old; cerebral hemorrhage; FOUR score: 3; GCS: 3.

**Table 3:** Characteristics of the 120 patients with brain-damage

Statistics/category	Frequency
Age (years), mean ± SD	47.9 ± 14.8
Gender, male, n (%)	85 (70.8 %)
Diagnosis, n (%)	
TBI	53 (44.2 %)
Intracerebral hemorrhage	38 (31.7 %)
Subarachnoid hemorrhage	22 (18.3 %)
Brain tumor	3 (2.5 %)
Hydrocephalus	2 (1.7 %)
Epilepsy	1 (0.8 %)
Cerebral infarction	1 (0.8 %)
Outcome, n (%)	
Good outcome (MRS, 0-2)	42 (35 %)
Poor outcome (MRS, 3-6)	78 (65 %)
In-hospital death	26 (21.7 %)
Consciousness, n (%)	
Alert	33 (27.5 %)
Drowsy	28 (23.3 %)
Stuporous	44 (36.7 %)
Comatose	15 (12.5 %)

*TBI* traumatic brain injury

### FOUR and GCS scores

**Fig. 2:**
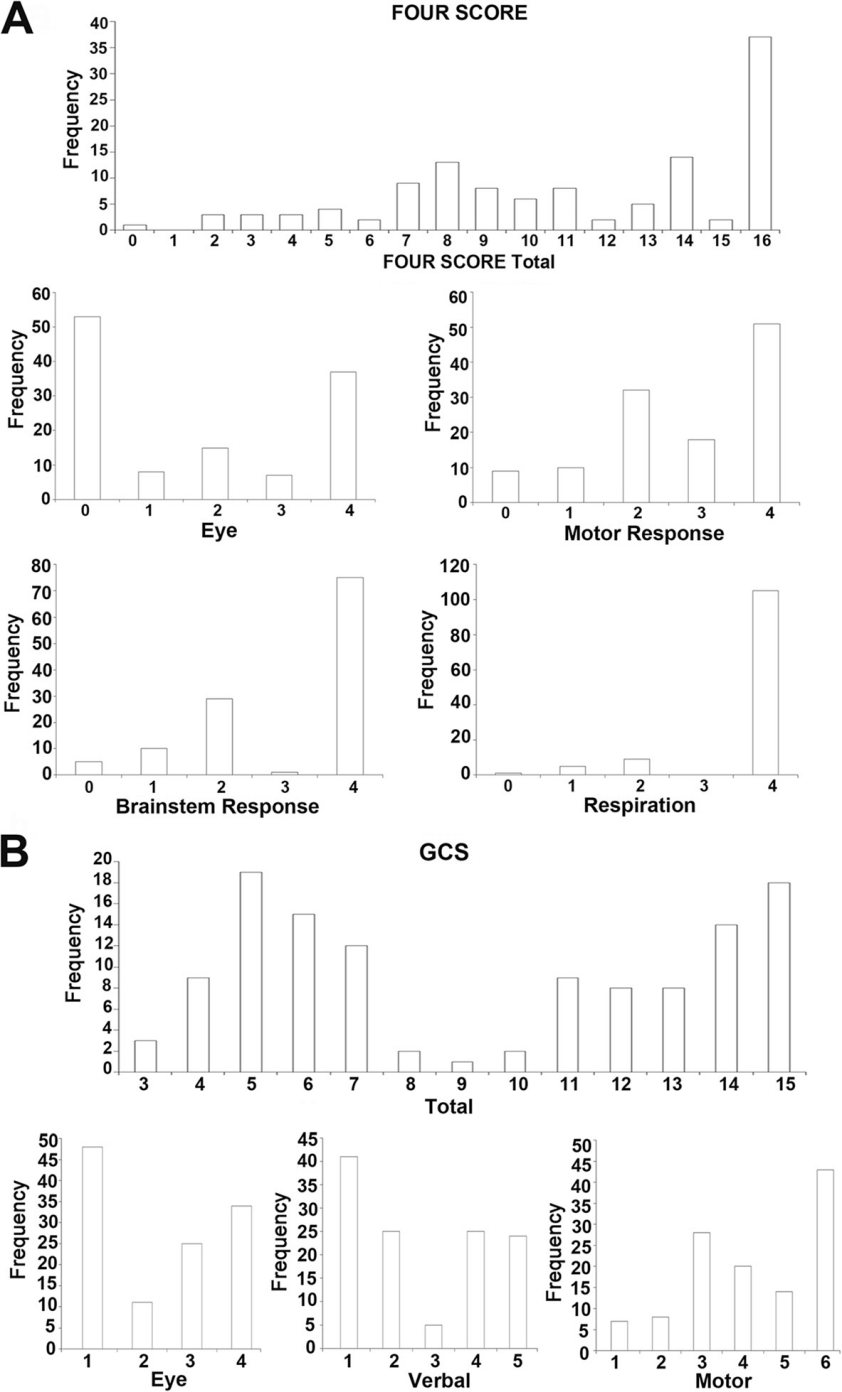
**a** Distribution of the Full Outline of Unresponsiveness (FOUR); **b** Glasgow Coma Scale (GCS), and their respective sub-scores

The distributions of the patients according to FOUR scores and GCS are shown in Fig. 2. For the FOUR score (discrete distribution, non-normally distributed), the maximum grade of 16 was the most represented among the patients, corroborating the results obtained for motor response, respiration, and brain stem response; the majority of patients had an eye sub-score of 0. In the case of GCS (discrete distribution, non-normally distributed), the distribution was rather sparse, with 5, 6, 7, 14, and 15 being the leading overall scores (Fig. 2). Interestingly, the overall reliability was excellent for the FOUR and the GCS scores (Table 4). In addition, the FOUR score yielded an excellent test-retest reliability (ICC = 0.930; *p* < 0.001) (Table 4).

**Table 4:** Intraclass correlation coefficient (ICC) for the FOUR score and the Glasgow Coma Scale for different raters

Rater Pair	No. of patients	FOUR score					GCS			
		Eye	Motor	Brainstem	Respiration	Total	Eye	Motor	Verbal	Total
R/R	40	0.988	0.993	0.928	0.987	0.991	0.958	0.934	0.961	0.971
N/N	47	0.928	0.968	0.919	0.955	0.973	0.933	0.894	0.893	0.949
N/R	33	0.764	0.939	0.779	0.895	0.929	0.906	0.831	0.863	0.940
0veral	120	0.932	0.970	0.885	0.941	0.970	0.939	0.900	0.914	0.958

*FOUR* Full Outline of Unresponsiveness, *GCS* Glasgow Coma Scale, *N* nurse, *R* resident

### Intraclass correlation

For the FOUR score, intraclass correlation coefficients (ICC) for the alert, drowsy, stuporous, and comatose groups were 0.888 (95 % CI, 0.776–0.945), 0.696 (95 % CI, 0.351–0.859), 0.891 (95 % CI, 0.801–0.940), and 0.879 (95 % CI, 0.649–0.959), respectively. For GCS, ICC values for the alert, drowsy, stuporous, and comatose groups were 0.712 (95 % CI, 0.422–0.857), 0.761 (95 % CI, 0.489–0.889), 0.521 (95 % CI, 0.126–0.738), and 0.696 (95 % CI, 0.122–0.897), respectively. The FOUR score had a slightly higher inter-observer agreement for the diagnosis of traumatic head injury compared with GCS. The overall ICC of the FOUR score for the traumatic and non-traumatic head injury groups were 0.977 (95 % CI, 0.959–0.986) and 0.964 (95 % CI, 0.941–0.978), respectively. ICC of the GCS for the traumatic and non-traumatic head injury groups were 0.956 (95 % CI, 0.924–0.975) and 0.959 (95 % CI, 0.934–0.975), respectively. The overall ICC for the FOUR score for the intubated and non-intubated patients were 0.940 (0.899–0.965) and 0.956 (0.927–0.973), respectively. The overall ICC for the GCS score for the intubated and non-intubated patients were 0.858 (0.760–0.916) and 0.953 (0.922–0.972), respectively (Table 5).

**Table 5:** Intraclass correlation coefficient (ICC) for the FOUR score and the Glasgow Coma Scale in different patients

Patients (n)	FOUR score (95 % CI)	GCS (95 % CI)
Consciousness		
Alert (33)	0.888 (0.776–0.945)	0.712 (0.422–0.857)
Drowsy (28)	0.696 (0.351–0.859)	0.761 (0.489–0.889)
Stuporous (44)	0.891 (0.801–0.940)	0.521 (0.126–0.738)
Comatose (15)	0.879 (0.649–0.959)	0.696 (0.122–0.897)
Diagnosis		
TBI (53)	0.977 (0.959–0.986)	0.956 (0.924–0.975)
Non-TBI (67)	0.964 (0.941–0.978)	0.959 (0.934–0.975)
Intubation		
Intubated (58)	0.940 (0.899–0.965)	0.858 (0.760–0.916)
Non-intubated (62)	0.956 (0.927–0.973)	0.953 (0.922–0.972)

### Consistency

The Cronbach’s α showed a high degree of internal consistency for the FOUR score (first rating, α = 0.846; second rating, α = 0.844; all *p* < 0.001) and the GCS (first rating, α = 0.916; second rating, α = 0.904; all *p* < 0.001). Spearman’s correlation coefficients between the GCS and FOUR scores were high and statistically significant (first rating, *r* = 0.932; second rating, *r* = 0.887; *p* < 0.001).

### Prognosis

**Fig. 3:**
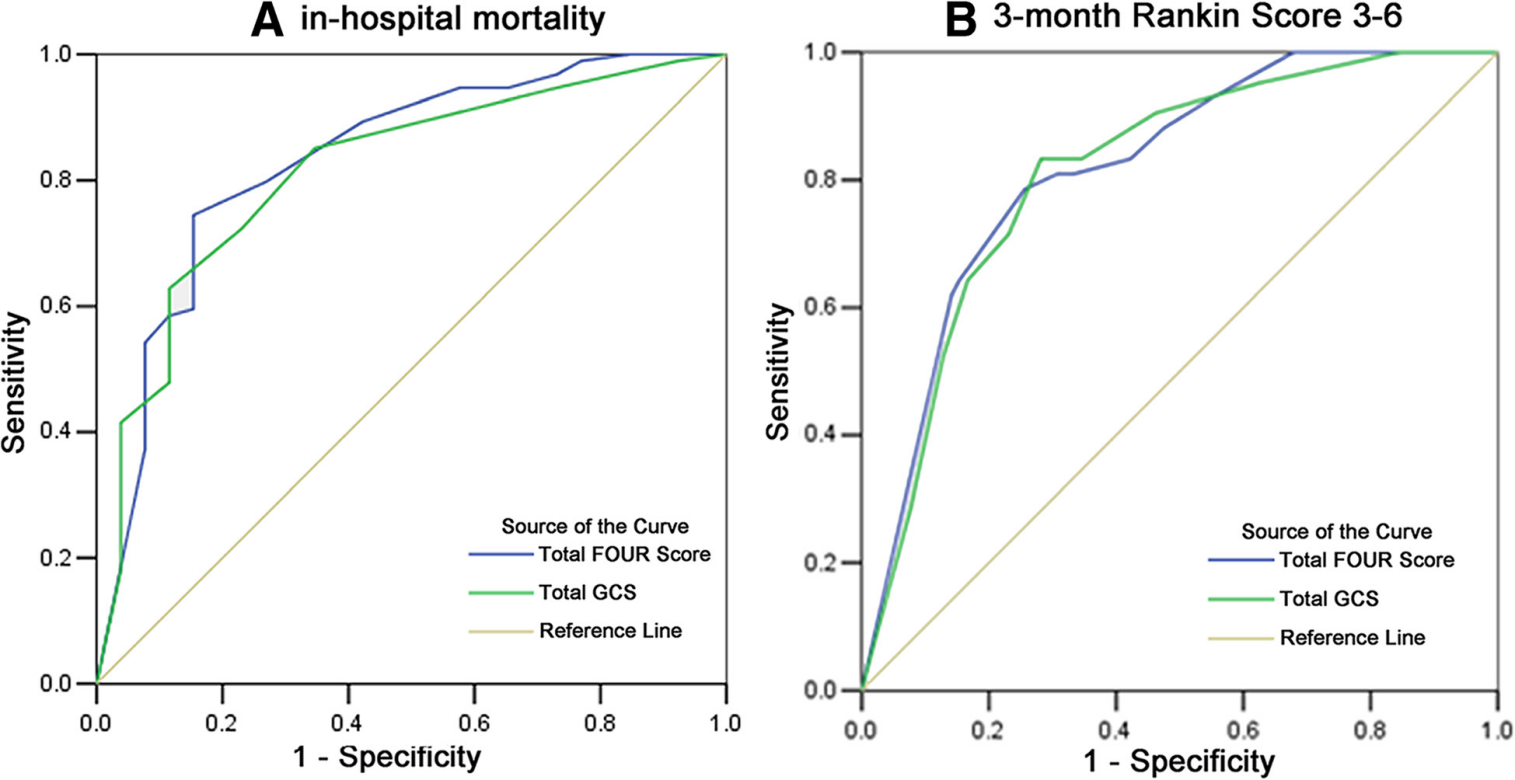
**a** Receiver operating characteristic (ROC) curves comparing Full Outline of Unresponsiveness (FOUR) score and Glasgow Coma Scale (GCS) in predicting in-hospital mortality; **b** Receiver operating characteristic (ROC) curves comparing Full Outline of Unresponsiveness (FOUR) score and Glasgow Coma Scale (GCS) in predicting 3-month MRS 3-6

Regarding in-hospital mortality (Fig. 3), areas under the curve (AUC) for the FOUR and GCS scores were 0.834 (95 % CI 0.740-0.928) and 0.815 (95 % CI 0.723–0.908), respectively. The maximized scores predicting in-hospital mortality were 9 for the FOUR score (sensitivity, 75 %; specificity, 85 %) and 7 for the GCS (sensitivity, 63 %; specificity, 89 %). Similarly, as shown in Fig. 3, the FOUR score AUC for unfavorable outcome (MRS > 2) was higher compared to that obtained with the GCS (0.818 vs. 0.812). The optimal score predicting a poor outcome was 13 for the FOUR score (sensitivity, 79 %; specificity, 74 %) and 10 for the GCS (sensitivity, 83 %; specificity, 72 %). Considering the components of the FOUR and GCS scores, all sub-scores had good predictive values for poor outcome (MRS 3-6) except respiration (AUC = 0.596, 95 % CI 0.495–0.698) of the FOUR score. Table 6 displays various areas under the curve for each outcome.

**Table 6:** Receiver operating characteristic curve analyses in predicting MRS 3-6, hospital mortality for GCS, FOUR score, and subunits of two scales

Variable	Hospital mortality AUC (95 % CI)	MRS 3-6 AUC (95 % CI)
Total FOUR score	0.834 (0.740–0.928)	0.818 (0.742–0.894)
Eye	0.758 (0.661–0.855)	0.788 (0.699–0.877)
Motor	0.789 (0.681–0.896)	0.785 (0.701–0.869)
Brainstem reflexes	0.764 (0.656–0.872)	0.708 (0.616–0.799)
Respiration	0.692 (0.561–0.824)	0.596 (0.495–0.698)
Total GCS	0.815 (0.723–0.908)	0.812 (0.734–0.891)
Eye	0.771 (0.681–0.861)	0.786 (0.698–0.874)
Motor	0.731 (0.633–0.829)	0.796 (0.713–0.879)
Verbal	0.793 (0.689–0.897)	0.780 (0.697–0.863)

*AUC* area under the curve, *CI* confidence interval, *FOUR* Full Outline of Unresponsiveness, *GCS* Glasgow Coma Scale, *MRS* modified Rankin score

## Discussion

To date, many prospective studies have assessed the FOUR score, which is now widely used in the clinical setting. However, no Chinese version of the FOUR score was available. This study demonstrated that the Chinese version of the FOUR score has a good concurrent validity, a high degree of internal consistency, and a good inter-rater reliability among medical staff, and is at least as good as the GCS. These results are comparable to previous studies [10–14]. Inter-rater agreement ranges from good to excellent in all patient categories, showing a greater agreement compared with the GCS. For both the Chinese version of the FOUR score and the GCS score, the overall reliability of each rater pair was excellent, with intraclass correlations (ICC) of 0.929-0.991. The FOUR score showed a good test-retest reliability (ICC = 0.930; *p* < 0.001), suggesting that the Chinese version of the FOUR score is with high time-stability and consistency. The lowest ICC values were obtained by a rating pair comprising a resident and a nurse for both scales, but these values were still excellent for the Chinese version of the FOUR and GCS scores (0.929, 95 % CI, 0.857–0.965; 0.940, 95 % CI, 0.880–0.970, respectively). The results from this study are consistent with previous studies [13]. Of all the sub-scales in the Chinese version of the FOUR score, inter-rater agreement for the brainstem sub-scale was the lowest, especially in stuporous and comatose patients. Nevertheless, these values were still excellent (ICC = 0.885, 95 % CI, 0.835–0.920) and in line with previous studies [11, 28, 29], but inconsistent with Iyer et al. [10]. This may be explained by the fact that stuporous and comatose patients’ pupil and corneal reflexes are not sensitive enough, and it is not easy to distinguish when their mental status changes. Besides, other factors such as the time spent to observe pupils and corneal reflexes, measurement methods for the pupil sizes, and corneal reflexes, may differ.

Total scores of the FOUR and GCS scores were similar in predicting mortality and unfavorable outcome, in agreement with previous findings [17, 30]. Comparing the AUC of total GCS and total FOUR scores, the value for respiration patterns in the FOUR score was lowest. This may be explained by the fact that most patients were given a score of 4 (regular breathing pattern), with no patients having 3 (Cheyne-Stokes breathing), and few having 0 (breathes at ventilator rate or apnea). In this study, we validated a cut-off point of 9 for the Chinese version of the FOUR score, and 7 for the GCS in hospital mortality, which is in line with the inventor of the FOUR score and Okasha et al. [31]. In this study, nearly half of the patients were intubated, which was similar to previous studies [3, 29]. The FOUR score showed a good consistency for both the intubated and non-intubated patients, which was in agreement with the results reported by Kramer et al. [29]. These findings also suggest that the Chinese version of FOUR score may be used in multiple departments including intensive care unit and other units that use mechanical ventilation. However, prospective studies with large sample sizes are needed to validate these findings.

A few limitations of this study should be addressed. First, this was a single-site study with a relatively small sample size. We also enrolled few comatose patients. Further study assessing those particular patients is needed to better evaluate the scaling ystems. In addition, we only enrolled raters who had >10 years of clinical experience, while inexperienced medical staff were not included. Finally, we did not test the Chinese version of the FOUR score in child populations with head trauma, and did not stratify the analysis in TBI patients due to their small number.

## Conclusions

In conclusion, the Chinese version of the FOUR score can be used to reliably assess patients with impaired consciousness. The scaling system is easily taught and remembered, allows detection of locked-in syndromes as well as the presence of a vegetative state, and is useful to predict poor outcome. Based on these findings, the Chinese version of the FOUR score is a reliable tool for evaluating LOC in patients with brain damage, and worthy of recommendation and application in clinical practice.

## Abbreviations

AUC: areas under the curve
ESICM: European Society of Intensive Care Medicine
FOUR: Full Outline of Unresponsiveness
GCS: Glasgow Coma Scale
ICC: intraclass correlation coefficient
ICU: intensive care unit
LOC: level of consciousness
ROC: receiver operating characteristic
SD: standard deviation
TBI: traumatic brain injury
